# Immunohistochemical Expression of Platelet-Derived Growth Factor Receptor β (PDGFR-β) in Canine Cutaneous Peripheral Nerve Sheath Tumors: A Preliminary Study

**DOI:** 10.3390/vetsci9070345

**Published:** 2022-07-09

**Authors:** Catarina Aluai-Cunha, Augusto Matos, Irina Amorim, Fátima Carvalho, Alexandra Rêma, Andreia Santos

**Affiliations:** 1 Department of Veterinary Clinics, Institute of Biomedical Sciences Abel Salazar (ICBAS), University of Porto, R. Jorge Viterbo Ferreira 228, 4050-313 Porto, Portugal; up201507059@up.pt (C.A.-C.); ajmatos@icbas.up.pt (A.M.); 2 Animal Science and Study Centre (CECA), Food and Agrarian Sciences and Technologies Institute (ICETA), P. Gomes Teixeira, Apartado 55142, 4051-401 Porto, Portugal; 3 Department of Pathology and Molecular Immunology, Institute of Biomedical Sciences Abel Salazar (ICBAS), University of Porto, R. Jorge Viterbo Ferreira 228, 4050-313 Porto, Portugal; ifamorim@icbas.up.pt (I.A.); mdfaria@icbas.up.pt (F.C.); airema@icbas.up.pt (A.R.); 4 Institute of Molecular Pathology and Immunology (IPATIMUP), University of Porto, R. Júlio Amaral de Carvalho 45, 4200-135 Porto, Portugal; 5 Institute for Research and Innovation in Health (I3S), University of Porto, R. Alfredo Allen 208, 4200-135 Porto, Portugal

**Keywords:** dog, Ki-67, PDGFR-β, PNST, sarcomas

## Abstract

**Simple Summary:**

The peripheral nerve sheath tumors are relatively common neoplasms, belong to the soft tissue sarcomas group, and are poorly investigated in veterinary medicine; the diagnosis is complex, and therapeutic options are limited. The platelet-derived growth factor receptors, namely the β subunit, are an important class of tyrosine kinase receptors that can be activated by genetic alterations and contribute to the process of carcinogenesis, so the inhibition of this receptor is an important therapeutic target. Using the immunohistochemical technique, this study aims to evaluate the expression of this receptor in 19 samples, 10 malignant and 9 benign tumors. The results showed that the majority of benign tumors, about 67% of cases, expressed the receptor in less than 25% of neoplastic cells and, in 80% cases of malignant tumors, the receptor was expressed in more than 25% of neoplastic cells. It was also found that, in the larger tumors, the expression of this receptor was significantly higher. With these findings it seems reasonable to speculate that the drugs able to inhibit this receptor, such as toceranib, may be considered in the therapeutic approach of these tumors.

**Abstract:**

As in humans, the prevalence of tumors in companion animals is increasing dramatically and there is a strong need for research on new pharmacological agents particularly for the treatment of those tumors that are resistant to conventional chemotherapy agents such as soft tissue sarcomas (STS). Because malignant (MPNST) and benign peripheral nerve sheath tumors (BPNST) are relatively common STS in dogs, the aim of this retrospective study was to evaluate the immunohistochemical (IHC) expression of PDGFR-β, contributing to its characterization as a potential target for their treatment. A total of 19 samples were included, 9 histologically classified as benign and the other 10 as malignant. The results showed diffuse immunoexpression in the cytoplasm of neoplastic cells. Six (66.7%) BPNST expressed the receptor in less than 25% of neoplastic cells and only three (33.3%) exhibited labelling in more than 25% of neoplastic cells. In contrast, all MPNST expressed PDGFR-β, and in 8 (80%) of these samples, the receptor was expressed in more than 25% of neoplastic cells, and only 2 (20%) cases expressed the receptor in less than 25% of neoplastic cells. PDGFR-β expression was significantly higher in MPNST and larger tumors, suggesting that drugs able to inhibit the activity of this tyrosine kinase receptor, such as toceranib, may be considered in the approach of unresectable tumors and/or in the context of adjuvant or neoadjuvant therapies.

## 1. Introduction

Soft tissue sarcomas (STS) are tumors derived from tissues of mesenchymal origin, meaning they can arise from almost any anatomical site [1,2,3,4]. They are a heterogeneous population constituting 15% and 7% of all cutaneous and subcutaneous tumors in dogs and cats, respectively [5]. Most STS are single masses that appear in middle-aged to elderly dogs, with no racial or gender predisposition, but with a greater tendency to affect large breeds [5]. Clinical signs are directly related to the location and size of the tumor, but in most cases, animals are asymptomatic [5]. STS larger than 5 cm and located at the distal limbs, histological grade III and those excised with incomplete margins are considered to bear poor prognosis [5]. In such cases, the use of post-operative adjuvant therapies is recommended, including radiotherapy to control local disease and chemotherapy to control distant metastatic disease.

Malignant peripheral nerve sheath tumors (MPNST) are a subtype of STS arising from Schwann cells, perineural cells or endoneural and perineural fibroblasts and they account for 27% of all peripheral nervous system tumors in dogs [6,7]. They are microscopically non-encapsulated, consist of usually highly pleomorphic cells with numerous mitotic figures, have an infiltrative growth pattern and metastasize to distant organs [8,9,10]. PNST may also present a benign behavior and the most common benign PNST (BPNST) are schwannomas and neurofibromas, which are well circumscribed and located in the skin or subcutaneous tissue [11]. Both types of PNST are reported in different species [12].

Platelet-derived growth factor receptors (PDGFRs) α and β are tyrosine kinase type receptors (TKR) that participate in cellular physiological and pathological signaling pathways, mainly through paracrine mechanisms. In the adult physiological processes, they stimulate fibroblast and endothelial cell proliferation and are involved in tissue regeneration and fibrosis [13,14]. These receptors activate many signal transduction pathways, including PI3K, MAPK and phospholipase Cγ and AKT [15] that stimulate proliferation and cell growth and control the expression of anti-apoptotic genes [16,17,18].

However, PDGFRs can be aberrantly activated by several genetic alterations contributing to carcinogenesis [19,20]. Neoplasms of mesenchymal, glial and hematopoietic origins reveal dysfunctions of PDGFR [21]. The most frequent alterations are overexpression, constitutive activation of the tyrosine kinase domain, and post-transcriptional regulation by specific RNA sequences such as miRNA-34 [22].

In human medicine, PNST have been found to express specifically PDGFR-β so therapies targeting this receptor may have therapeutic potential [23,24]. In veterinary medicine, PDGFR-β expression has been investigated in canine osteosarcomas [25], nasal carcinomas [26], transitional cell carcinomas of the bladder [27], anal sac gland adenocarcinomas [28], oral melanomas [29], mammary carcinomas [30], liposarcomas [31], perivascular wall tumors [32], hemangiosarcomas and hemangiomas [33].

To the best of our knowledge, PDGFR-β has not been studied in canine PNST, and therefore, the goal of this preliminary study was to evaluate the PDGFR-β expression in these skin tumors through an immunohistochemical (IHC) assay, aiming to pave the way to investigate new potential therapeutic targets for effective management of patients affected by these tumors.

## 2. Materials and Methods

### 2.1. Samples and Histopathology

This study was conducted using archived samples from the Veterinary Pathology Laboratory of the University of Porto, after approval by the Ethical Committee for the Animal Wellbeing (ORBEA/P314/2019). Nineteen archived formalin-fixed paraffin-embedded (FFPE), Hematoxylin and Eosin (HE)-stained canine PNST tissue samples were selected, including 10 malignant cases (9 malignant schwannomas and one neurofibrosarcoma) and 9 benign schwannomas. All cases met the current diagnostic criteria [12,34,35,36], based on the evidence of typical histological features, such as the presence of specific growth patterns denominated Antoni A, characterized by well-defined cellular component, and Antoni B, characterized by hypocellular areas with loose fibrous to myxoid stroma and confirmed by immunohistochemical study with vimentin, S-100, NSE, GFAP and desmin; the immunostaining for all cases were positive for vimentin, S-100, NSE and GFAP, and negative for desmin. Clinical data regarding age, gender, breed, tumor size, location and margins were obtained from the histopathology reports. Breeds were grouped as miniature, small, medium, large and giant, according to the American Kennel Club standards. The neoplasm location was classified in two groups, limbs or trunk, and tumor size was categorized as <3 cm or ≥3 cm, using the longest tumor axis. Surgical margins were recorded and classified, accordingly to the pathologist as complete, close and/or incomplete. The pathologist considered complete margins whenever a distance between normal tissue and tumor cells were higher than 5 mm, close within 1–5 mm and if less than 1 mm it was considered infiltrated margins.

### 2.2. Immunohistochemistry (IHC)

Two µm thick tissue sections were subjected to IHC technique using a polymer-based system (Novocastra NovoLink-Max Polymer Detection System^TM^; Novocastra Laboratories, Newcastle, UK), according to the manufacturer’ instructions. Sections were dewaxed in xylene and rehydrated through graded alcohols. The antigen retrieval was performed using microwave radiation with Extran^®^ solution (at 1:50 dilution) for approximately 10 min. Then, slides were cooled and washed in Tris-buffered saline (TBS, pH 7.6, 0.5 M). Endogenous peroxidase was blocked with the addition of Peroxidase Block solution (Novocastra™) for 12 min at room temperature. To inhibit the binding of non-specific proteins, the Protein Block solution (Novocastra ™) was added for 1 h, at room temperature. Slides were then incubated overnight at 4 °C with a rabbit monoclonal anti-PDGFR-β antibody (clone 28E1, Cell Signaling Technology, Danvers, MA, USA), diluted 1:50 in TBS with 5% bovine serum albumin (BSA). After washing the slides with TBS, the Post Primary solution (Novocastra™) was added for 30 min at room temperature, followed by the addition of the Polymer solution (Novolink™) for another 30 min. After washing with TBS, slides were incubated with DAB Substrate Buffer (Novolink™) and DAB Chromogen (Novocastra™). Finally, they were washed for 10 min and contrasted with hematoxylin. Positive controls consisted of sections from canine hemangiosarcoma and the immunostaining of macrophages, fibroblasts and vascular smooth muscle cells were used as internal positive controls [37]. Negative controls comprised sections in which the primary antibody was replaced by buffer solution. The cross reactivity of the primary antibody for canine tissues has been previously validated by Asa et al. (2012; 2013) [33,38].

Tumor cells were considered positive when there was distinct brown cytoplasmatic immunolabelling and tumors were grouped on the basis of the estimated percentage of immunoreactive cells (weakly positive from 0 to 25% and strongly positive if >25%) according to a previously described method [33,38]. The labelling intensity was classified into 2 groups: weak or intense.

The Ki-67 IHC method was performed as previously described by Teixeira et al. (2016) [39] using a monoclonal antibody (MIB-1, Dako, Glostrup, Denmark, 1:500). The proliferation index (PI) was defined as the percentage of immunopositive nuclei, determined by counting 1000 nuclei in the hot spot regions (×400). For statistical analysis, tumors were grouped according to the percentage of positive cells as <20% or ≥20%, a cut-off value previously defined by Watanabe et al. (2001) [40].

Slides were examined independently by three observers (C.A.C., I.A. and A.S.) and, in case of disagreement, a consensual result was reached through simultaneous evaluation of the slide using a multi-head microscope.

### 2.3. Statistical Analysis

The results were analyzed with the SPSS software (version 26.0; SPSS Inc., Statistical Products and Services, Chicago, IL, USA). For statistical analysis, tumors were grouped according to a cut-off value of 25% PDGFR-β positive cells. The relationship between PDGFR-β expression and clinical data, such as gender, tumor location (trunk or limbs), size (<3 cm and ≥3 cm) and breed (small versus medium + large + giant), as well as with histopathological factors, such as tumor type (BPNST vs. MPNST) and proliferation index (Ki-67 < 20% or ≥20%) were analyzed by Fisher’s exact test. ANOVA analysis was used to compare the mean Ki-67 index with the type of neoplasia and the expression of PDGFR-β. Values of p < 0.05 were considered statistically significant.

## 3. Results

Data regarding breed, age, gender, type of tumor, tumor location, dimensions, Ki-67 proliferation index and % of PDGFR-β expression are summarized in Table 1.

The mean age ± SD of the animals with benign neoplasms was 10.67 ± 2.35 years while those with malignant neoplasms were 9.00 ± 3.23 years old. In this population, the PNST occurrence was similar between males (10 cases—52.6%), and females (9 cases—47.4%), although the latter presented a higher number of malignant neoplasms (6/9) when compared to males (4/10).

Most of PNST was found in larger breed animals (11/14), and the majority of them were malignant (Table 2). All small breed dogs presented a benign PNST. Regarding surgical margins, 15 cases were incompletely removed (8 benign and 7 malignant), 9 in the limbs and 4 in the trunk (missing information in 2 cases).

Tumor behavior was independent of tumor location, but a significant association was found between MPNST and higher tumor sizes (Table 2); all MPNST had ≥3 cm. Although not statistically significant, MPNST had a slightly higher mean Ki-67 index (26.01 ± 20.09) than BPNST (15.66 ± 20.32) (Table 3). The relationship between the mean of Ki-67 index and PDGFR-β expression was also not statistically significant (Table 3).

**Figure 1:**
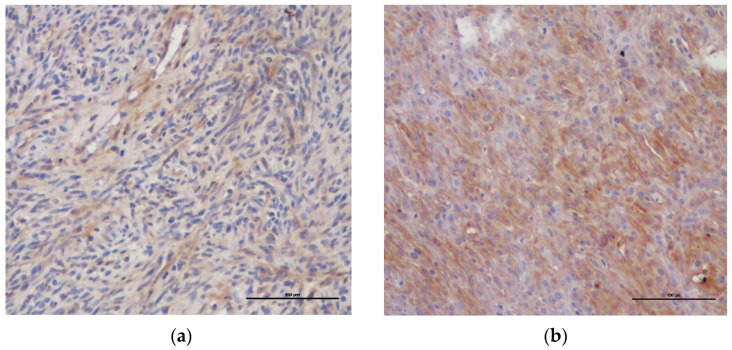
Immunohistochemical PDGFR-β staining in canine cutaneous PNST (×200): (**a**) MPNST with weak cytoplasmic staining in more than 25% of neoplastic cells; (**b**) MPNST with intense cytoplasmic staining in more than 25% of neoplastic cells.

**Figure 2:**
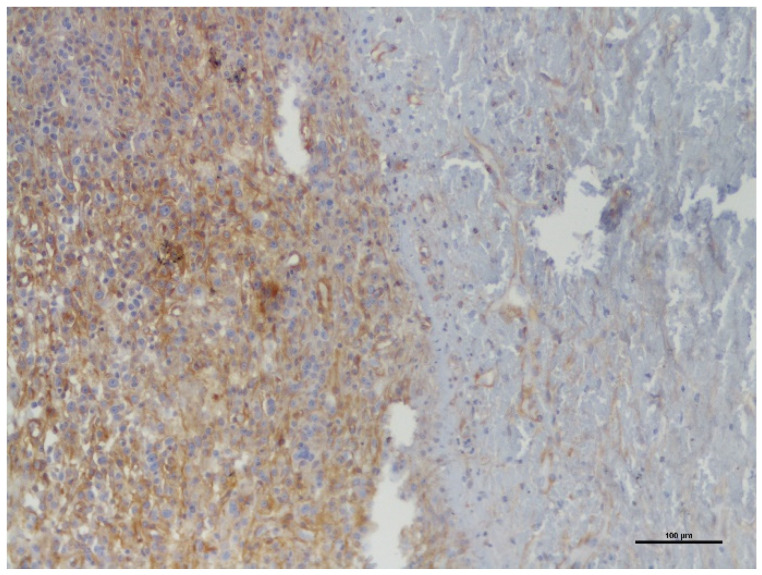
Immunohistochemical staining of PDGFR-β in canine cutaneous MPNST (×100). Tumor invasive front showing high levels of PDGFR-β expression.

The PDGFR-β staining was diffuse cytoplasmatic in both benign and malignant PNST. The labelling intensity of BPNST was weak in 8 cases and intense in 1 case; in MPNST, it was weak in 6 cases and intense in 4 cases (Figure 1a,b). In addition, MPNST showed a more intense PDGFR-β expression at the tumor invasive front and areas of higher cytological atypia (Figure 2).

PDGFR-β was expressed in less than 25% of tumor cells in 6 of 9 BPNST samples (66.7%), including 1 case without immunoreactivity. All MPNST expressed PDGFR-β, with 80% of them (8/10) in >25% of cells. PDGFR-β expression was significantly higher in MPNST when compared to BPNST (Table 4). The tumor location, tumor size and Ki-67 index were not significantly related with the levels of PDGFR-β expression (Table 4).

## 4. Discussion

The activation of PDGFRs has an important role in the carcinogenesis of some human and animal neoplasms [19,20]. In dogs, the expression of PDGFR-β has been better characterized in hemangiosarcomas where it seems to contribute directly to a malignant behavior [33]. Several studies have shown that other canine neoplasms express this TKR suggesting that it may constitute an important therapeutic target [23,25,26,27,28,29,30,31,32,33,41]. To date, there are no published studies on the expression of this receptor in animal PNST, although it has already been studied in human PNST [24].

Despite the small sample in this preliminary study, it was possible to verify that the age of development of PNST does not appear to be related to their biological behavior, which is in accordance with the existing literature [5]. It has been described that STS appear in middle-aged to elderly dogs, and in large breeds, with no gender predisposition [2,3,5,42]. In this study, the prevalence of PNST was higher in large breed dogs, corroborating the previously described data [2,3,5]. The Ki-67 index and means were not significantly different between benign or malignant PNST and were also not related with the levels of PDGFR-β expression. The small number of cases and the great variability of Ki-67 values among them (which included two benign cases with very high Ki-67 index and two malignant with low Ki-67) may justify these results. Therefore, the value of Ki-67 as a proliferation marker to discriminate the clinical behavior (benign versus malignant) of theses tumors and the influence of PDGFR-β for tumor proliferation should be evaluated in larger series.

BPNST showed a significant lower expression of PDGFR-β compared to MPNST, suggesting that the receptor may have a role in the malignant transformation of these neoplasms, as also has been demonstrated in human PNST [23]. It has been reported that PDGFRs have a role in chemokine signaling to the tumor microenvironment and stimulate stromal cells contributing to mesenchymal carcinogenesis [43].

Larger tumors exhibited a higher level of expression of this TKR, suggesting that it may also contribute to tumor growth and perhaps a worse prognosis, but survival studies are needed to confirm this hypothesis. In addition, malignant PNST showed a more intense PDGFR-β expression at the tumor invasive front and areas of higher cytological atypia, suggesting that it may contribute to the tumor local invasive capacities [13,14,16,17,18].

Considering the overexpression of PDGFR-β in MPNST and in larger tumors, it seems reasonable to speculate that tyrosine kinase inhibitors (TKI) such as toceranib (Palladia^®^), may have a role in the treatment of the more aggressive PNST. In fact, the blockage of PDGFR-β expression with imatinib, a TKI used in human medicine, decreases the epithelial-mesenchymal transition (EMT) and the transcription of factor Slug, and it dramatically reduces the progression of human sarcomas [44].

This preliminary investigation had the strong limitation of the low number of cases, which limits the statistical power of the results, and therefore, further studies with a large number of PNST samples are warranted. Although PNST are not very common, they may have different clinical behaviors, and their proper diagnosis should be emphasized. The diagnostic process includes a panel of immunohistochemical markers that are expensive to perform and, because of that, many of these tumors are diagnosed only as cutaneous sarcomas and not distinguished from other soft tissue sarcomas, which may have consequences in terms of prognosis and therapeutic approach.

## 5. Conclusions

In summary, this study described, for the first time, the PDGFR-β expression in canine PNST, evidencing its presence in the cytoplasm of neoplastic cells. Despite the small number of samples, it was demonstrated that malignant and larger PNST have a significantly higher level of this TKR, suggesting that the receptor may have a role in the pathogenesis and progression of these neoplasms. These preliminary results support the need for conducting more investigations and survival studies to understand the value of PDGFR-β at both prognostic and therapeutic levels. Considering that some of these tumors may be difficult to totally excise, they may require adjuvant or neoadjuvant treatments, justifying the need for new therapeutic strategies, such as toceranib, a veterinary TKI able to target PDGFR-β.

## Figures and Tables

**Table 1:** Clinical data, type of tumor, Ki-67 index and percentage of PDGFR-β expression in dogs with cutaneous PNST.

Case Nr.	Breed	Age (Years)	Gender	Type of Tumor	Location of Tumor	Dimensions of Tumor (cm)	Ki-67 PI (%)	% PDGFR-β Expression
1	M. Schnauzer	13	M	B	T	2.5 × 2.5	60.8	≤25%
2	Mixed breed	12	F	B	L	4 × 3.5	15.8	>25%
3	Poodle	9	M	B	NR	3 × 2.5	36.3	>25%
4	X Rottweiler	8	M	B	NR	NR	4.3	≤25%
5	Mixed breed	8	M	B	L	4 × 2.8	1.8	≤25%
6	Mixed breed	10	F	B	L	2 × 1	3.8	≤25%
7	G. Shepherd	14	F	B	T	4 × 2	14.1	≤25%
8	Poodle	13	M	B	L	1.2 × 1.2	1.2	≤25%
9	Boxer	9	M	B	T	2 × 1	2.8	>25%
10	Port. Pointer	9	M	M	T	8 × 8	38.9	>25%
11	St. Bernard	10	F	M	L	10 × 10	62.6	≤25%
12	Siberian husky	13	F	M	L	4.5 × 4	14.1	>25%
13	Labr. Retriever	2	M	M	T	3 × 3	23.5	>25%
14	Boxer	7	M	M	L	4.5 × 3.5	26.2	>25%
15	Boxer	7	F	M	T	6.5 × 6	21.4	>25%
16	Mixed breed	12	M	M	L	3 × 2	54.0	≤25%
17	Mixed breed	12	F	M	T	10.5 × 8	6.4	>25%
18	Dalmatian	10	F	M	L	4 × 2.5	10.2	>25%
19	Boxer	8	F	M	L	4 × 2	2.8	>25%

M—Male; F—Female; B—BPNST; M—MPNST; L—Limbs; T—Trunk; PI—Proliferation index.

**Table 2:** Relationship between tumor type and clinicopathological variables in canine PNST.

Variable	Nr. of Tumors	BPNST (Number; %)	MPNST (Number; %)	*p* Value
Gender				
Female	9	3 (33.3)	6 (66.7)	0.24
Male	10	6 (60.0)	4 (40.0)	
Breed *				
Small	3	3 (100.0)	0 (0)	0.05
Medium, Large, Giant	11	3 (27.3)	8 (72.7)	
Tumor Location *				
Trunk	7	3 (42.9)	4 (57.1)	0.64
Limbs	10	4 (40.0)	6 (60.0)	
Tumor size *				
<3 cm	4	4 (100.0)	0 (0)	0.02
≥3 cm	14	4 (28.6)	10 (71.4)	

Fields with * have a lower number of cases due to unknown data. Group differences were assessed by Fisher’s exact test.

**Table 3:** Relationship between the mean Ki-67 expression, tumor type and PDGFR-β expression in canine PNST.

Variable	Nr. of Tumors	Mean Ki-67	Standard Deviation (SD)	*p* Value (ANOVA)
Tumor Type				
Benign	9	15.66	20.32	0.28
Malignant	10	26.01	20.09	
PDGFR-β expression				
≤25%	8	25.33	28.38	0.45
>25%	11	18.04	12.48	

**Table 4:** Relationship between PDGFR-β expression and clinicopathological variables in 19 canine PNST.

Variable	Nr. of Tumors	Tumors with ≤25% PDGFR-β Expression (Number; %)	Tumors with >25% PDGFR-β Expression (Number; %)	*p* Value
Tumor type				
Benign	9	6 (66.7)	3 (33.3)	0.05
Malignant	10	2 (20.0)	8 (80.0)	
Tumor Location *				
Trunk	7	2 (28.6)	5 (71.4)	0.35
Limbs	10	5 (50.0)	5 (50.0)	
Tumor size *				
<3 cm	4	3 (75.0)	1 (25.0)	0.13
≥3 cm	14	4 (28.6)	10 (71.4)	
Ki-67 Index				
<20%	11	5 (45.5)	6 (54.5)	0.55
≥20%	8	3 (37.5)	5 (62.5)	

Fields with * have a lower number of cases due to unknown data. Group differences were assessed by Fisher’s exact test.

## Data Availability

The data that support the findings of this study are available from the corresponding author upon reasonable request.
